# Allelopathic effects of *Aizoon canariense* leaf leachates on growth, biochemistry, and oxidative stress responses in selected crop species

**DOI:** 10.3389/fpls.2025.1659978

**Published:** 2025-09-02

**Authors:** Suliman M. S. Alghanem

**Affiliations:** Department of Biology, College of Science, Qassim University, Burydah, Saudi Arabia

**Keywords:** *Aizoon canariense*, allelopathy, leaf leachate, oxidative stress, antioxidant enzymes

## Abstract

**Introduction:**

Allelopathy offers a promising ecological approach to sustainable weed and crop management, yet the allelopathic potential of many underutilized wild species remains unexplored. *Aizoon canariense*, a xerophytic plant native to arid regions, may produce bioactive compounds capable of influencing the growth and physiology of neighboring crops. This study aimed to evaluate the chemical composition and allelopathic effects of *A. canariense* aqueous leaf leachates (LL) on four major crops—wheat (*Triticum aestivum*), barley (*Hordeum vulgare*), rapeseed (*Brassica napus*), and mung bean (*Vigna radiata*).

**Methods:**

The chemical constituents of *A. canariense* leaves were identified using gas chromatography–mass spectrometry (GC-MS). Four crops were treated with 10% and 15% LL, and various morphological (shoot/root length, biomass), physiological (chlorophyll, carotenoids), and biochemical parameters (total phenols, flavonoids, DPPH activity, SOD, CAT, H₂O₂, and MDA content) were evaluated. Correlation analysis was performed to assess relationships among stress responses and growth indicators.

**Results and Discussion:**

GC-MS analysis revealed 40 compounds, including sesquiterpenes, phytol, patchouli alcohol, and α-cadinol. LL treatments significantly reduced shoot and root growth, pigment content, and biomass in a dose-dependent manner, with rapeseed and mung bean showing the greatest sensitivity. Conversely, LL exposure elevated phenolic and flavonoid levels, antioxidant enzyme activity (SOD, CAT), and oxidative stress markers (H₂O₂ and MDA). Correlation analysis demonstrated strong negative relationships between growth traits and oxidative damage, and positive associations between phenolic accumulation and antioxidant responses. *A. canariense* leaf leachates exert significant allelopathic effects on crop plants by inducing oxidative stress and activating antioxidant defenses, suggesting the presence of potent phytotoxic compounds. These findings offer new insights into the ecological role of *A. canariense* and its potential application as a natural bioherbicide or rotation crop component in sustainable agriculture.

## 1 Introduction

Modern agriculture faces a growing challenge in maintaining crop productivity while minimizing reliance on synthetic agrochemicals. One critical but often underutilized approach to sustainable crop management is the study and application of allelopathy—where plants release chemical compounds (allelochemicals) into the environment that influence the growth and development of other organisms (Cheng and Cheng, 2015; Kong et al., 2024). Allelopathic interactions play a vital role in shaping plant community dynamics, influencing weed suppression, crop rotation success, and soil microbial ecology (Mushtaq and Fauconnier, 2024).

Allelochemicals are typically secondary metabolites such as phenolics, flavonoids, terpenoids, alkaloids, and organic acids. These compounds can be released into the environment through various plant parts, including roots, leaves, stems, seeds, and decaying litter, often via leaching, volatilization, or root exudation (Choudhary et al., 2023; Scavo et al., 2019). When present in the soil or rhizosphere, they may disrupt multiple physiological processes in neighboring plants, including seed germination, root elongation, photosynthesis, nutrient uptake, and enzyme activity (Choudhary et al., 2023; Scavo et al., 2019). At the cellular level, allelochemicals can induce oxidative stress by generating reactive oxygen species (ROS), which damage membranes, nucleic acids, and proteins, often reflected in elevated levels of lipid peroxidation and stress signaling compounds like hydrogen peroxide (H_2_O_2_) and malondialdehyde (MDA) (Bogatek and Gniazdowska, 2007; Gniazdowska et al., 2015).

*A. canariense*, a member of the family Aizoaceae, is a xerophytic species distributed in arid and semi-arid regions. Despite its presence in drought-prone habitats and reports of ethnobotanical uses, the allelopathic potential of *A. canariense* remains largely unexplored (Osman et al., 2024). Xerophytic plants such as *A. canariense* often accumulate high levels of bioactive compounds, potentially as an adaptive response to environmental stress (Ibrahim et al., 2022; Mohanta et al., 2024). These compounds may confer strong allelopathic effects when introduced into the soil environment via leachates or decomposition, influencing the physiological processes of neighboring cultivated species.

Previous studies have highlighted that allelopathic stress can impair key physiological functions in crops, such as chlorophyll and carotenoid synthesis (Kumar et al., 2024a), and can activate antioxidative defense systems including enzymes like superoxide dismutase (SOD) and catalase (CAT) (Šoln et al., 2022).Plants may also respond by modulating levels of phenolic compounds and flavonoids, which play dual roles in antioxidant defense and stress signaling (Saini et al., 2024). These changes serve as biochemical markers of allelopathic interference and can help differentiate between tolerance and susceptibility among plant species (Al-Qthanin et al., 2024).

Given the ecological and agronomic significance of allelopathy, particularly from underutilized wild plants, it is crucial to investigate how their bioactive compounds influence the growth and metabolism of common crop species (Pedrol and Puig, 2024; Vajja et al., 2025). Despite its presence in arid ecosystems and potential medicinal properties, *A. canariense* has not been systematically studied for its allelopathic effects. This study is novel in its integrative approach—linking detailed phytochemical profiling via GC-MS with physiological, biochemical, and oxidative stress responses in key crops. By focusing on both primary (growth and biomass) and secondary (antioxidant and stress marker) responses, the research not only highlights the potential phytotoxicity of *A. canariense* but also its possible role as a natural source of growth modulators or allelochemicals. Such findings are particularly relevant in the context of sustainable agriculture, where natural plant-based compounds could serve as eco-friendly alternatives to synthetic agrochemicals.

Therefore, the objectives of this study were to: (i) characterize the chemical composition of *A. canariense* leaf extract using GC-MS; (ii) evaluate the allelopathic effects of its aqueous leaf leachate (LL) at two concentrations (10% and 15%) on the growth, physiological attributes, and biochemical responses of four economically important crops—wheat (*Triticum aestivum*), barley (*Hordeum vulgare*), rapeseed (*Brassica napus*), and mung bean (*Vigna radiata*); and (iii) explore the relationships among morphological traits, antioxidant responses, and oxidative stress indicators using correlation analysis. The findings from this study are expected to advance understanding of the allelopathic potential of *A. canariense*, support its ecological characterization, and explore its possible application in natural weed suppression or crop rotation strategies. Furthermore, the identification of specific allelochemicals and their biological effects may provide a scientific basis for future development of *A. canariense*-derived bioproducts in sustainable crop production systems.

## 2 Materials and methods

### 2.1 Plant material collection and processing of *A. canariense* leaves

Fresh, healthy leaves of *A. canariense* were collected from a natural population in The Qassim Region (Al-Qassim Province) in Saudi Arabia, more broadly, the Qassim region lies between latitudes 24° 41′ N and 27° 19′ N and longitudes 41° 38′ E and 44° 50′ E, and thoroughly rinsed with distilled water to remove dust and debris. The cleaned leaves were blot-dried and oven-dried at 70 °C for 48 hours. Dried material was then ground into a fine powder using a laboratory grinder and stored in airtight containers at room temperature until further use.

### 2.2 Preparation of leaf leachate

To prepare the leachates, 10 g and 15 g of powdered *A. canariense* leaves were added separately to 100 mL of distilled water in Erlenmeyer flasks. The mixtures were allowed to soak at room temperature (25 ± 2 °C) for 48 hours with intermittent shaking. After incubation, suspensions were filtered using Whatman No. 1 filter paper and centrifuged at 3000 rpm for 10 minutes to remove particulates. The final volume was adjusted to 100 mL using distilled water.

### 2.3 pH and electrical conductivity of LL

The pH and EC of each leachate were recorded prior to application. The pH values were 6.14 (10% LL) and 5.87 (15% LL), while EC values were 1.72 dS/m (10% LL) and 2.31 dS/m (15% LL), respectively. To distinguish allelochemical effects from abiotic stress due to pH and osmotic potential, a pH/EC-matched control (using distilled water adjusted with dilute HCl and NaCl) was included. This matched control enabled us to isolate the chemical (allelochemical) effects from those potentially caused by altered pH or ionic strength. All leachates were stored at 4 °C and used within 48 hours of preparation to minimize degradation of bioactive compounds (Khalediyan et al., 2021).

### 2.4 GC-MS analysis of LL extracts

The chemical composition of *A. canariense* leaf extracts was analyzed using gas chromatography–mass spectrometry (GC-MS) on an Agilent 7890A GC system coupled with a 5975C inert mass selective detector (MSD). The separation was performed using an HP-5MS capillary column (30 m × 0.25 mm i.d., 0.25 µm film thickness). The GC oven temperature was initially set at 50 °C (held for 2 min), then ramped to 150 °C at 10 °C/min, followed by an increase to 280 °C at 5 °C/min, and held for 10 minutes. The injector temperature was set at 250 °C. Helium was used as the carrier gas at a constant flow rate of 1.0 mL/min. The injection volume was 1 µL in splitless mode. The mass spectrometer was operated in electron impact (EI) mode at 70 eV, with a scan range of 50–550 m/z. Compounds were tentatively identified by comparing the acquired mass spectra with those from the NIST (National Institute of Standards and Technology) library database, and confirmed where possible by comparison with authentic standards and retention indices.

### 2.5 Experimental design

Seeds of four crop species—wheat, barley, rapeseed, and mung bean—were obtained from a certified local seed supplier. The seeds were surface-sterilized using 5% sodium hypochlorite solution for 5 minutes, thoroughly rinsed with distilled water, and blot-dried on sterile filter paper. Sterilized seeds were sown in plastic pots (15 cm diameter) containing a sterilized mixture of sand and loamy soil in a 3:1 ratio (v/v). All pots received 250 mL of full-strength Hoagland nutrient solution at sowing to ensure uniform nutrient availability during early seedling development.

After germination, five healthy and uniform seedlings were maintained in each pot. For the first seven days, seedlings were irrigated with Hoagland solution to support initial establishment. Thereafter, plants were subjected to three treatment regimes: (a) control (standard Hoagland solution), (b) 10% *A. canariense* leaf leachate (LL), and (c) 15% LL. In the LL treatments, the Hoagland nutrient solution was prepared by dissolving all its constituents directly into the respective concentrations of LL, replacing distilled water to maintain nutrient consistency across treatments. The concentrations of 10% and 15% were selected based on preliminary screening trials, which revealed these levels as biologically effective in eliciting allelopathic responses without causing complete growth inhibition or seedling mortality. Lower concentrations showed negligible effects, while higher levels were excessively phytotoxic. This approach ensures a meaningful comparison of stress responses while maintaining plant viability, and aligns with previous studies on allelopathic aqueous extracts.

The treatments were applied on alternate days for ten consecutive days, with each pot receiving 50 mL of the respective treatment solution per application. Each treatment was replicated three times following a completely randomized design (CRD) under greenhouse conditions maintained at 25 ± 2 °C with a 12 h light/12 h dark photoperiod. At 17 days after sowing (i.e., after 15 days of LL treatment), plants were carefully uprooted, washed with distilled water to remove soil particles, and immediately processed for morphological and biochemical analyses as described in subsequent sections.

### 2.6 Growth parameters

After 15 days of treatment, shoot length and root length were measured using a ruler. Fresh weights of shoots and roots were recorded immediately after harvest. Plant tissues were then oven-dried at 70 °C for 48 h to obtain shoot dry weight (DW) and root dry weight.

### 2.7 Total chlorophyll and carotenoid content

Total chlorophyll and carotenoid contents in the leaves of treated and control plants were determined spectrophotometrically following the method of Wellburn (1994) with slight modifications. Fresh leaf tissue (0.1 g) was homogenized in 5 mL of 80% (v/v) chilled acetone using a pre-cooled mortar and pestle. The homogenate was centrifuged at 10,000 rpm for 10 min at 4°C to separate the pigment-containing supernatant from plant debris (Wellburn, 1994).

The absorbance of the clear supernatant was measured at 645 nm, 663 nm, and 470 nm using a UV–Visible spectrophotometer (Shimadzu UV-1800, Japan). Pigment concentrations were calculated using the following equations (Lichtenthaler and Wellburn, 1983):


$$\text{Total chlorophyll}\left({\text{mg g}}^{-1}\text{FW}\right)=\left(20\mathrm{.2 \times A645}\right)+\left(8\mathrm{.02 \times A663}\right)$$

$$\text{Carotenoids }\left({\text{mg g}}^{\mathrm{-1}}\text{FW}\right)\text{ = }\left(1000 \times A470 – 1\mathrm{.82 \times Chl a – 85}.02 \times Chl b\right)/198$$

All measurements were performed in triplicate, and results were expressed as milligrams of pigment per gram of fresh weight (mg g^-1^ FW). To ensure accuracy, blank samples containing 80% acetone were used for baseline calibration. Proper care was taken to perform all procedures under dim light or in the dark to prevent pigment degradation.

### 2.8 Total phenolic content

The total phenolic content of leaf tissues was quantified using the Folin–Ciocalteu colorimetric method, as described by Singleton and Rossi (1965), with slight modifications. Approximately 0.5 g of fresh leaf tissue was homogenized in 5 mL of 80% (v/v) methanol using a chilled mortar and pestle (Singleton and Rossi, 1965). The homogenate was centrifuged at 10,000 rpm for 10 minutes at 4 °C, and the supernatant was collected for further analysis. To quantify phenolic compounds, 0.5 mL of the methanolic extract was mixed with 2.5 mL of 10% (v/v) Folin–Ciocalteu reagent. After 5 minutes, 2.0 mL of 7.5% (w/v) sodium carbonate solution was added to the mixture. The reaction mixture was vortexed briefly and then incubated in the dark at room temperature for 30 minutes to allow for complete color development.

The absorbance of the resulting blue-colored solution was measured at 765 nm using a UV-Vis spectrophotometer against a blank containing all reagents except the plant extract. Gallic acid was used as a standard for the calibration curve, and results were expressed as milligrams of gallic acid equivalents (mg GAE) per gram of fresh weight (FW).

### 2.9 Total flavonoid content

Total flavonoid content in the leaf samples was determined using the aluminum chloride colorimetric method, following the protocol of Shraim et al. (2021) with slight modifications. Fresh leaf tissue (0.5 g) was homogenized in 5 mL of 80% methanol, and the homogenate was centrifuged at 10,000 rpm for 10 minutes. The resulting supernatant was used as the crude extract for flavonoid quantification (Shraim et al., 2021).

For the assay, 0.5 mL of the methanolic leaf extract was mixed with 0.1 mL of 10% (w/v) aluminum chloride, 0.1 mL of 1 M potassium acetate, and 4.3 mL of 80% methanol in a test tube. The reaction mixture was thoroughly mixed and incubated at room temperature for 30 minutes in the dark to allow complex formation. The absorbance of the resulting yellow-colored complex was measured at 415 nm using a UV-Visible spectrophotometer against a reagent blank. A standard calibration curve was prepared using known concentrations of quercetin, and total flavonoid content was expressed as milligrams of quercetin equivalents (mg QE) per gram of fresh weight (FW).

### 2.10 Hydrogen peroxide content

H_2_O_2_ content in leaf tissues was quantified following the method of Velikova et al. (2000), with minor modifications. Fresh tissue (0.5 g) was homogenized in 5 mL of chilled 0.1% (w/v) trichloroacetic acid (TCA) using a pre-cooled mortar and pestle. The homogenate was centrifuged at 12,000 rpm for 15 minutes at 4 °C, and the clear supernatant was collected for analysis (Velikova et al., 2000). For the assay, 0.5 mL of the supernatant was mixed with 0.5 mL of 10 mM potassium phosphate buffer (pH 7.0) and 1 mL of 1 M potassium iodide (KI). The mixture was incubated at room temperature for 15 minutes in the dark to allow the reaction to proceed. The absorbance of the reaction mixture was then measured at 390 nm using a UV-Visible spectrophotometer. H_2_O_2_ content was quantified using a standard curve prepared with known concentrations of H_2_O_2_ and expressed as micromoles per gram of fresh weight (µmol g^-1^ FW H_2_O_2_).

### 2.11 Lipid peroxidation (MDA content)

Malondialdehyde (MDA) content, a key indicator of lipid peroxidation and oxidative membrane damage, was estimated using the thiobarbituric acid (TBA) reaction assay as described by Heath and Packer (1968), with slight modifications. Fresh leaf tissue (0.5 g) was homogenized in 5 mL of 0.1% (w/v) trichloroacetic acid (TCA) using a chilled mortar and pestle. The homogenate was centrifuged at 12,000 rpm for 15 minutes at 4 °C, and the supernatant was carefully collected (Heath and Packer, 1968). To 1 mL of the supernatant, 4 mL of 0.5% (w/v) thiobarbituric acid prepared in 20% TCA was added. The mixture was vortexed and then heated at 95 °C in a water bath for 30 minutes. After incubation, the tubes were immediately cooled in an ice bath to stop the reaction and centrifuged again at 10,000 rpm for 10 minutes to remove any particulate matter.

The absorbance of the clear supernatant was measured at 532 nm and corrected for nonspecific turbidity by subtracting the absorbance at 600 nm. The MDA concentration was calculated using an extinction coefficient of 155 mM^-1^ cm^-1^ and expressed as micromoles of MDA per gram of fresh weight (µmol MDA g^-1^ FW). All samples were analyzed in triplicate to ensure reproducibility.

### 2.12 Antioxidant activity (DPPH assay)

The antioxidant activity of the tested leaf extracts was evaluated using the 2,2-diphenyl-1-picrylhydrazyl (DPPH) free radical scavenging assay, following the protocol of Shraim et al. (2021) with slight modifications (Shraim et al., 2021). Initially, 24 mg of DPPH were dissolved in 100 mL of methanol to prepare a stock solution. This solution was then filtered using methanol to obtain a working solution with an absorbance of approximately 0.973 at 517 nm. For the assay, 3 mL of the DPPH working solution was mixed with 100 µL of the methanolic plant leaf extract in test tubes. A control solution was prepared by adding 100 µL of methanol instead of extract to 3 mL of the DPPH solution. All tubes were incubated in complete darkness at room temperature for 30 minutes to prevent photodegradation of DPPH. After incubation, the absorbance was measured at 517 nm using a UV–Vis spectrophotometer. The radical scavenging activity (RSA) percentage was calculated using the following formula:


DPPH scavenging activity (%) = (A0-As/A0) ×100

Where: A0 = absorbance of the control (DPPH + methanol), As = absorbance of the sample (DPPH + plant extract).

### 2.13 Superoxide dismutase activity

Superoxide dismutase (SOD; EC 1.15.1.1) activity was determined based on its ability to inhibit the photochemical reduction of nitroblue tetrazolium (NBT), following the method of Beauchamp and Fridovich (1971) with slight modifications. Fresh leaf tissue (0.5 g) was homogenized in 5 mL of ice-cold 50 mM phosphate buffer (pH 7.8) containing 1% polyvinylpyrrolidone (PVP), and the homogenate was centrifuged at 12,000 rpm for 15 minutes at 4 °C. The resulting supernatant was used as the crude enzyme extract (Beauchamp and Fridovich, 1971).

The reaction mixture for SOD assay consisted of 50 mM phosphate buffer (pH 7.8), 13 mM methionine, 75 µM NBT, 2 µM riboflavin, 0.1 mM EDTA, and 0.1 mL of enzyme extract in a final volume of 3 mL. The reaction tubes were illuminated under fluorescent light for 15 minutes to initiate NBT photoreduction, while identical tubes kept in the dark served as blanks. The reduction of NBT to blue formazan was monitored by measuring absorbance at 560 nm using a UV-Visible spectrophotometer. One unit of SOD activity was defined as the amount of enzyme required to cause 50% inhibition of NBT reduction under the assay conditions. Results were expressed as enzyme activity units per gram of fresh weight (U g^-1^ FW). All samples were assayed in triplicate, and enzyme extracts were maintained on ice throughout the procedure to preserve enzymatic activity.

### 2.14 Catalase activity

Catalase (CAT; EC 1.11.1.6) activity was determined by monitoring the decomposition of H_2_O_2_ at 240 nm, following the method of Aebi (1984) with slight modifications. Fresh leaf tissue (0.5 g) was homogenized in 5 mL of ice-cold 50 mM phosphate buffer (pH 7.0), and the homogenate was centrifuged at 12,000 rpm for 15 minutes at 4 °C. The resulting supernatant was used as the crude enzyme extract for the assay (Aebi, 1984).

The reaction mixture consisted of 2.5 mL of 50 mM phosphate buffer (pH 7.0), 0.4 mL of freshly prepared 15 mM H_2_O_2_ solution, and 0.1 mL of enzyme extract, bringing the total volume to 3.0 mL. The decrease in absorbance, indicating the decomposition of H_2_O_2_, was recorded at 240 nm for 1 minute using a UV-Visible spectrophotometer at 25 °C. Catalase activity was calculated using the molar extinction coefficient of H_2_O_2_ (ϵ = 39.4 mM^-1^ cm^-1^) and expressed as micromoles of H_2_O_2_ decomposed per minute per gram of fresh weight (µmol H_2_O_2_ min^-1^ g^-1^ FW).

### 2.15 Statistical analysis

All experiments were conducted using three independent biological replicates, with each replicate consisting of a separate pot containing five uniform seedlings of a given species under identical treatment conditions (control, 10% LL, or 15% LL). All measurements and biochemical assays were performed using tissue sampled from individual plants, not pooled, to ensure accuracy and consistency. For each parameter, mean values were calculated across these three biological replicates (n = 3) and are reported as mean ± standard error (SE). Statistical analysis was performed using IBM SPSS Statistics version 26.0. One-way ANOVA was employed to test for significant differences among treatments for each species. When a significant effect was detected (p ≤ 0.05), the Least Significant Difference (LSD) test was used as a *post hoc* comparison method. Pearson’s correlation coefficients (r) were also calculated to explore the relationships among physiological (shoot/root traits), biochemical (phenols, flavonoids, DPPH, chlorophyll, carotenoids), and oxidative stress parameters (H_2_O_2_, MDA, SOD, CAT).

## 3 Results

### 3.1 GC-MS analysis of *A. canariense* leaf extract

GC-MS analysis of the leaf extract from *A. canariense* revealed the presence of a complex mixture of 40 volatile compounds, representing a wide range of chemical classes including sesquiterpenes, hydrocarbons, alcohols, ketones, and esters. The major constituents identified in the extract were patchouli alcohol (15.56%), α-cadinol (9.48%), tetratriacontane (8.70%), and e-cadinene (8.15%), indicating a predominance of sesquiterpene alcohols and hydrocarbons. Other notable compounds included dotriacontane (7.11%), 6-epi-shyobunol (3.30%), cubedol (3.73%), and phytol (2.29%) (**Table 1**).

**Table 1:** Chemical compounds detected and identified in *Aizoon* leaves by GCMS technique.

S. No	Compound name	Area %	RT	Confirmed by
1.	Azulene	0.75	18.52	STD
2.	Alpha-Guaiene	0.15	18.62	STD, MS
3.	α-Gurjunene	4.34	19.48	STD, MS
4.	Naphthalene	3.05	19.66	STD
5.	Patchoulene	1.27	20.15	STD, MS
6.	Caryophyllene	1.78	20.48	STD, MS
7.	Alloaromadendrene	1.04	21.52	STD
8.	Aromadendrene	1.44	21.55	MS, STD
9.	α- Muurolene	0.98	22.48	STD
10.	Amorphene	1.38	22.82	MS. STD
11.	c-cadinene	1.08	22.86	STD
12.	Naphthalene	0.02	22.89	MS, STD
13.	6-epi-shyobunol	3.30	22.97	RI, MS
14.	e-cadinene	8.15	23.06	STD
15.	Santalol	2.63	23.66	STD
16.	Cubedol	3.73	24.29	STD, MS
17.	Cubenol	0.94	25.19	RI
18.	Nerinine	1.06	25.54	STD
19.	2-(1-Cyclopent-1-enyl-1-methylethyl) cyclopentanone	0.07	25.54	STD
20.	Cedrenol	0.98	25.71	RI, MS
21.	Tetracyclo [6.3.2.0(2,5).0(1,8)] tridecan-9-ol, 4,4-dimethyl	4.40	25.82	STD
22.	Alpha-Eudesmol	1.01	26.01	STD, MS
23.	Junipene	0.01	26.01	STD
24.	Elemol	1.01	26.01	STD
25.	α-Cadinol	9.48	26.13	RI, MS
26.	Patchouli alcohol	15.56	26.99	STD
27.	7-Tetracyclo [6-2-1-0(3-8)0(3-9)] undecanol, 4,4,11,11-tetramethyl	1.18	27.20	STD
28.	i-Propyl5,8,11,14,17-eicosapentaenoate	1.92	28.87	STD
29.	6-Hydroperoxide-6-phenyl-5-carboxyethyl-2,3-dihydro-6H-pyran	1.07	29.24	STD
30.	Phytol	2.29	35.08	RI
31.	Cyclopentane, 1-(2-decyldodecyl)-2,4-dimethyl	0.86	37.14	STD
32.	Nantenine	0.90	38.86	STD
33.	Nonacosane	1.15	41.39	RI
34.	8’-O-Ethyl-á-Alectoronic acid	2.97	42.25	STD, MS
35.	Heptacosane	3.44	44.35	STD
36.	Tetratriacontane	8.70	47.15	RI, MS
37.	Dotriacontane	7.11	49.81	STD

RI, MS = conﬁrmed by n-alkanes retention index by mass spectra library; STD, MS=conﬁrmed by injection of standard and by mass spectra library; MS = tentative identiﬁcation=conﬁrmation only by mass spectra library.

**Figure 1:**
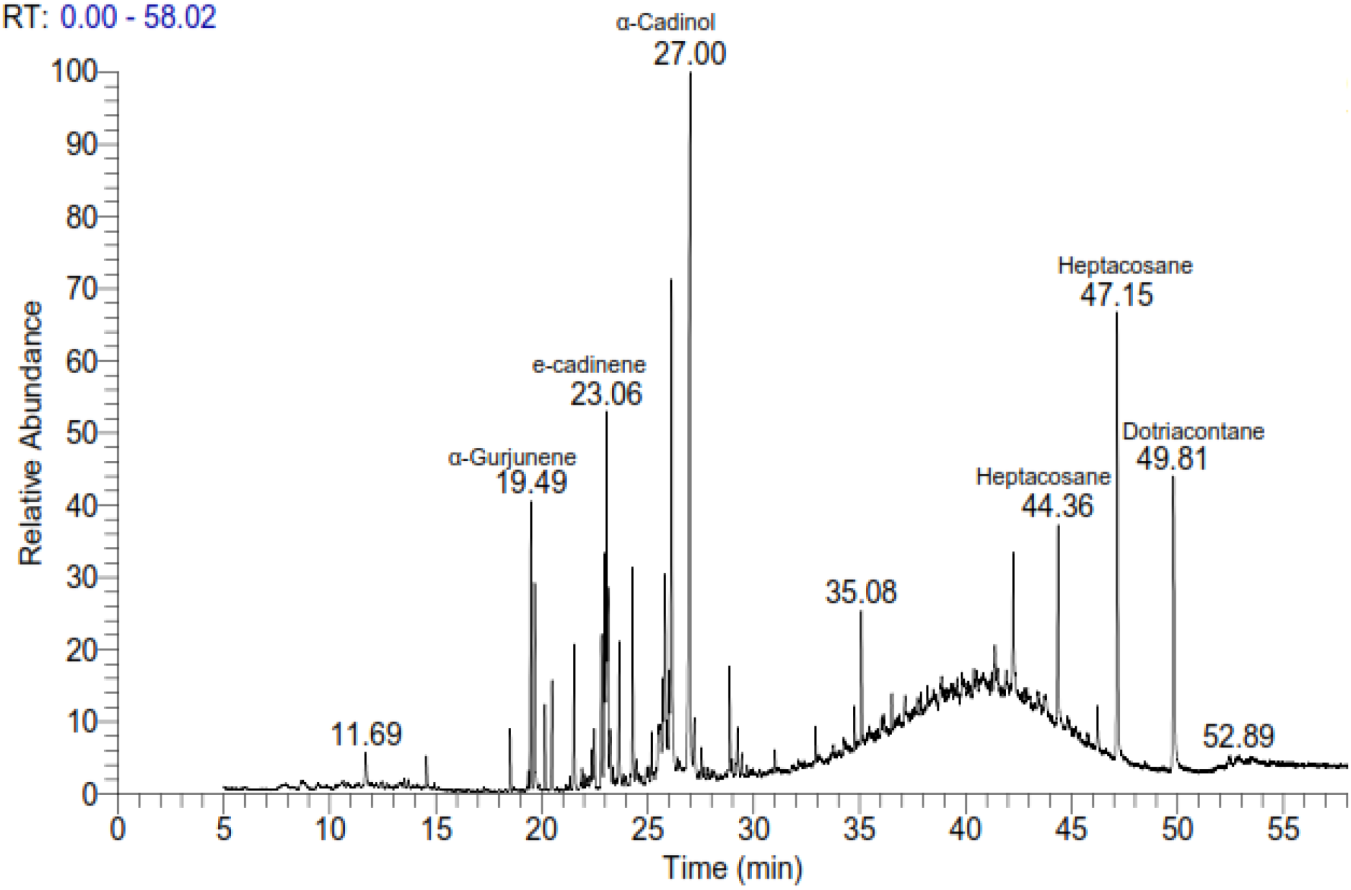
Gas chromatographic–mass spectrometric (GC-MS) chromatogram of extracts from *Aizoon canariense*. The chromatogram illustrates the chemical composition of the volatile compounds identified in the extract, based on their retention times and mass spectral data.

Naphthalene derivatives (two peaks at RT 19.66 and 22.89) were also detected, suggesting the presence of polycyclic aromatic hydrocarbons. Several oxygenated sesquiterpenes such as cubenol (0.94%), cedrenol (0.98%), and elemol (1.01%) were identified (**Figure 1**). The broad chemical diversity identified in the extract demonstrates the phytochemical richness of *A. canariense* and supports its potential for pharmacological and agronomic applications. The complete chromatogram is presented in **Figure 1**, with retention time and area percentage values summarized in **Table 1**.

### 3.2 Growth parameters

The application of *A. canariense* LL at 10% and 15% concentrations significantly inhibited the growth of all tested crop species—wheat, barley, rapeseed, and mung bean—in a concentration-dependent manner. Growth traits including shoot length, root length, and both shoot and root biomass showed clear declines compared to the control, with the degree of inhibition varying across species (**Table 2**).

**Table 2:** Effect of different concentration (10% and 15%) of leaf leachate (LL) of *Aizoon canariense* on shoot length, root length, shoot fresh weight, shoot dry weight, root fresh weight and root dry weight of wheat (*Triticum aestivum*), barley (*Hordeum* vulgare), rapeseed (*Brassica napus*) and mung bean (*Vigna radiata*). Data represent the mean ± standard error (SE) of three independent replicates. Data represent the means of three replications. Means sharing the same letter are not significantly different at the 5% probability level according to the Least Significant Difference (LSD) test. Inhibition percentages in parentheses (%).

Plant species	Treatments	Shoot length (cm)	Root length (cm)	Shoot FW (g)	Shoot DW (g)	Root FW (g)	Root DW (g)
Wheat	Control	20.2 ± 1.946a	10.16 ± 0.95a	1.067 ± 0.074a	0.2575 ± 0.074a	0.537 ± 0.082a	0.2638 ± 0.007a
	LL 10%	17.15 ± 0.54b (15.1%)	9.2 ± 0.1b (9.4%)	0.928 ± 0.0701b (13.0%)	0.1349 ± 0.0093b (47.6%)	0.43 ± 0.028b (19.9%)	0.1682 ± 0.021b (36.2%)
	LL 15%	13.2 ± 0.62c (34.7%)	7.97 ± 0.49c (21.6%)	0.8412 ± 0.032c (21.2%)	0.0791 ± 0.014c (69.3%)	0.2876 ± 0.047c (46.4%)	0.1213 ± 0.02c (54.0%)
>Barley	Control	18.0± 1.274a	9.63 ± 0.585a	0.9741 ± 0.026a	0.155 ± 0.054a	0.4997 ± 0.02a	0.2233 ± 0.014a
	LL 10%	15.3 ± 0.7ab (15.3%)	7.67 ± 1.001ab (20.4%)	0.925 ± 0.020ab (5.0%)	0.091 ± 0.0077ab (41.3%)	0.393 ± 0.019ab (21.4%)	0.1415 ± 0.013ab (36.6%)
	LL 15%	10.8 ± 0.781b (40.2%)	6.77 ± 0.288b (29.7%)	0.805 ± 0.019b (17.4%)	0.0824 ± 0.004b (46.8%)	0.2674 ± 0.013b (46.5%)	0.1113 ± 0.007b (50.2%)
>Rapeseed	Control	13.3 ± 0.62a	9.93 ± 0.152a	1.047 ± 0.067a	0.0869 ± 0.013a	0.4876 ± 0.022a	0.1979 ± 0.018a
	LL 10%	10.63 ± 1.02b (20.1%)	9.5 ± 0.264b (4.3%)	0.9631 ± 0.013b (8.0%)	0.0715 ± 0.001b (17.7%)	0.3683 ± 0.023b (24.5%)	0.1453 ± 0.017b (26.6%)
	LL 15%	7.6 ± 1.17c (42.9%)	8.73 ± 0.51c (12.1%)	0.8822 ± 0.014c (15.7%)	0.0532 ± 0.0061c (38.8%)	0.2336 ± 0.017c (52.1%)	0.1031 ± 0.018c (47.9%)
>Mung bean	Control	16.2 ± 1.708a	10.16 ± 0.907a	1.321 ± 0.08a	0.1069 ± 0.013a	0.604 ± 0.019a	0.3123 ± 0.019a
	LL 10%	13.8 ± 0.75ab (14.6%)	9.23 ± 0.472ab (9.2%)	1.129 ± 0.055ab (14.5%)	0.0994 ± 0.0036ab (7.0%)	0.4759 ± 0.04ab (21.2%)	0.2138 ± 0.013ab (31.5%)
	LL 15%	10.4 ± 0.72b (35.8%)	8.6 ± 0.321b (15.4%)	0.9595 ± 0.024b (27.4%)	0.08 ± 0.002b (25.2%)	0.4061 ± 0.006b (32.8%)	0.1593 ± 0.004b (49.0%)

In wheat, shoot length declined from 20.20 cm in the control to 17.15 cm (15.1% reduction) under 10% LL and to 13.20 cm (34.7% reduction) under 15% LL. Similarly, root length decreased by 18.2% and 42.3% under 10% and 15% LL, respectively. Shoot dry weight showed significant reductions, dropping from 0.2575 g in the control to 0.1427 g (44.6%) and 0.0791 g (69.3%) under 10% and 15% LL, respectively, highlighting the negative impact on biomass accumulation (**Table 2**).

Barley also exhibited strong sensitivity to LL treatment. Shoot length decreased by 24.4% and 40.2% under 10% and 15% LL, respectively, compared to the control value of 18.06 cm. Shoot DW was reduced by 41.8% under 10% LL and by 67.2% under 15% LL, indicating impaired shoot development. Root biomass also followed a similar declining trend.

Rapeseed appeared to be among the most sensitive species. Shoot length dropped from 13.30 cm in the control to 9.50 cm and 7.60 cm under 10% and 15% LL, corresponding to 28.6% and 42.9% reductions, respectively. Shoot DW was inhibited by 46.1% and 72.4%, while root DW declined by 39.3% and 65.7% under 10% and 15% LL treatments, respectively (**Table 2**).

Mung bean also showed considerable sensitivity to the treatments. Shoot length decreased from 16.20 cm in the control to 12.30 cm (24.1%) and 10.40 cm (35.8%) under 10% and 15% LL, respectively. Root DW declined by 35.6% and 49.8% across the two leachate concentrations. Shoot fresh weight followed a similar trend, indicating consistent growth suppression.

### 3.3 Chlorophyll and carotenoid content

**Figure 2:**
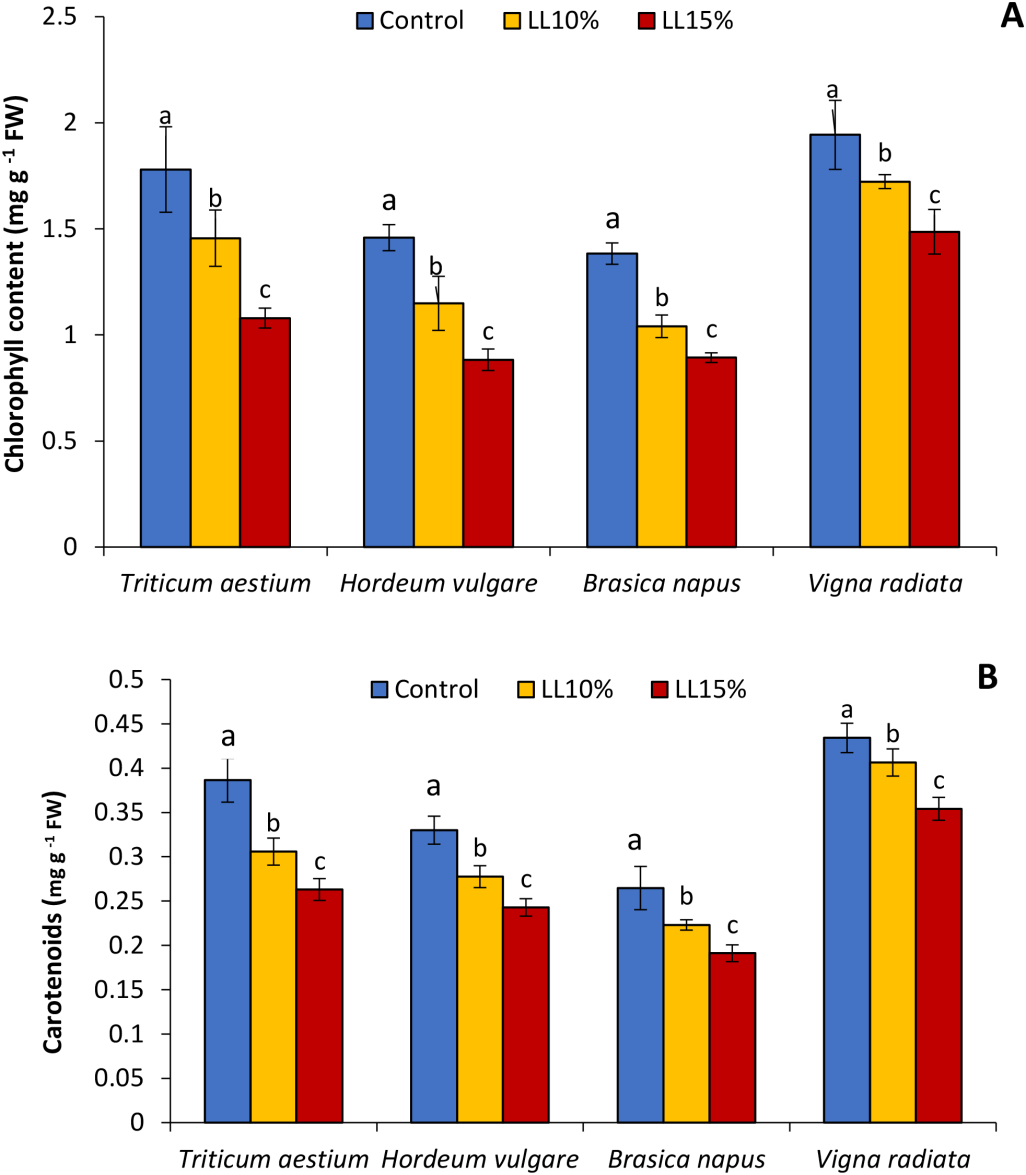
Effect of different concentrations (10% and 15%) of *Aizoon canariense* leaf leachate (LL) on **(A)** chlorophyll content and **(B)** carotenoid content in wheat (*Triticum aestivum*), barley (*Hordeum vulgare*), rapeseed (*Brassica napus*), and mung bean (*Vigna radiata*). Data represent the mean ± standard error (SE) of three independent replicates. Data represent the means of three replications. Means sharing the same letter are not significantly different at the 5% probability level according to the Least Significant Difference (LSD) test.

The application of *A. canariense* LL at 10% and 15% concentrations resulted in a concentration-dependent reduction in total chlorophyll and carotenoid contents in all four tested species—wheat, barley, rapeseed, and mung bean (**Figure 2**). Compared to the control, total chlorophyll content under 10% LL treatment decreased by 18.2% in wheat, 21.2% in barley, 24.8% in rapeseed, and 11.4% in mung bean. The inhibitory effect was more pronounced under the 15% LL treatment, with reductions of 39.4% in wheat, 39.5% in barley, 35.5% in rapeseed, and 23.5% in mung bean. These results indicate that photosynthetic pigment synthesis or stability was negatively impacted by LL, with rapeseed and barley being particularly sensitive at both concentrations (**Figure 2A**). Carotenoid content followed a similar trend (**Figure 2B**). The 10% LL treatment led to carotenoid reductions of 20.8% in wheat, 15.9% in barley, 15.7% in rapeseed, and 6.4% in mung bean. At 15% LL, the decreases were 31.9%, 26.5%, 27.8%, and 18.4%, respectively. Notably, wheat exhibited the greatest carotenoid inhibition, while mung bean showed the least reduction.

### 3.4 Phenolic and flavonoid content

**Figure 3:**
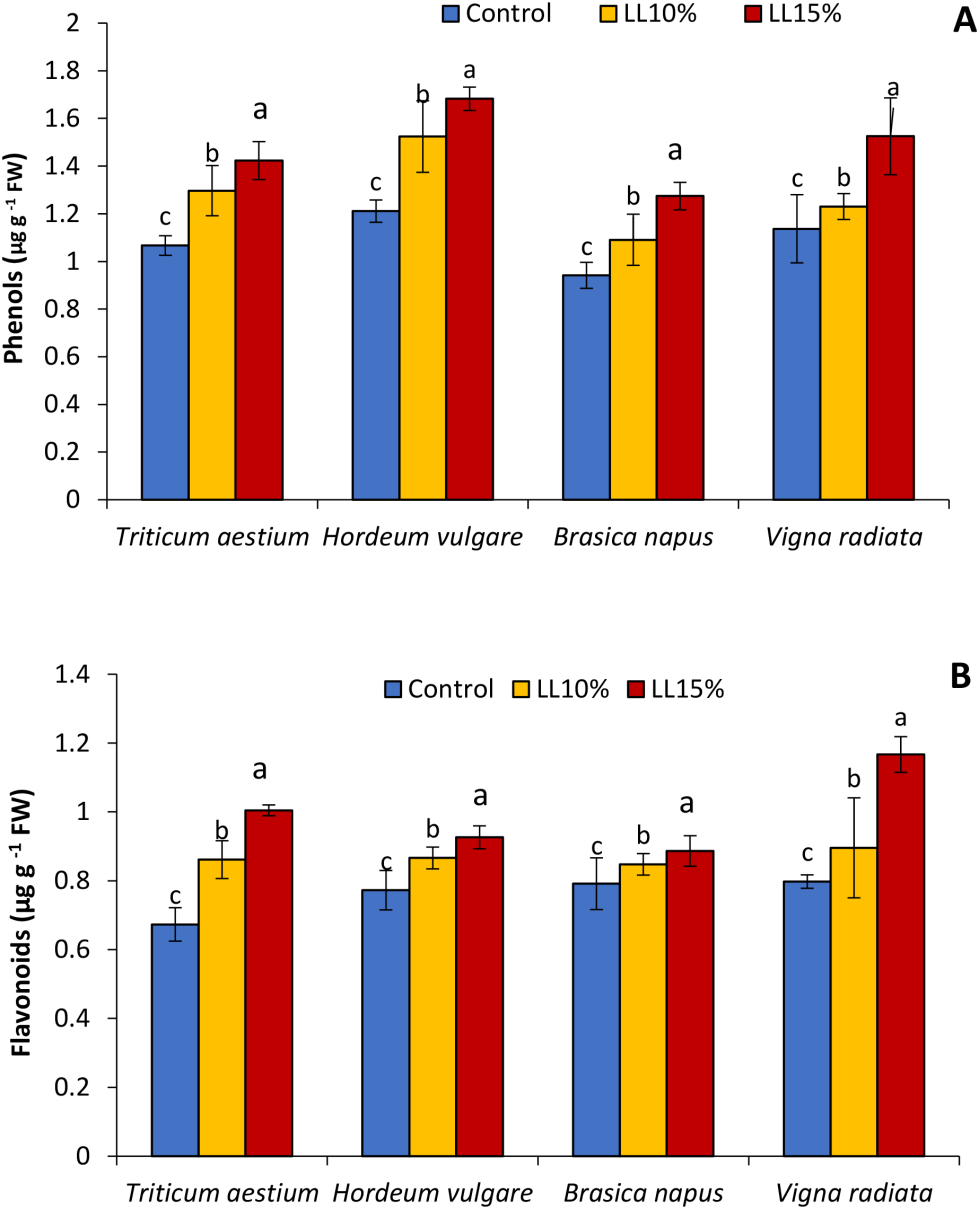
Effect of different concentrations (10% and 15%) of *Aizoon canariense* leaf leachate (LL) on **(A)** phenols content and **(B)** flavonoids content in wheat (*Triticum aestivum*), barley (*Hordeum vulgare*), rapeseed (*Brassica napus*), and mung bean (*Vigna radiata*). Data represent the mean ± standard error (SE) of three independent replicates. Data represent the means of three replications. Means sharing the same letter are not significantly different at the 5% probability level according to the Least Significant Difference (LSD) test.

The influence of *A. canariense* LL at 10% and 15% concentrations on phenolic and flavonoid contents in four crop species—wheat, barley, rapeseed, and mung bean—is illustrated in **Figure 3**. Total phenolic content (**Figure 3A**) increased significantly in all crops in response to LL treatments, with the 10% LL application resulting in increases of 21.6% in wheat, 25.8% in barley, 15.8% in rapeseed, and 8.2% in mung bean compared to controls. At 15% LL, these increases were more pronounced—33.4% in wheat, 38.9% in barley, 35.3% in rapeseed, and 34.1% in mung bean—suggesting a dose-dependent stimulation of phenolic biosynthesis. Flavonoid content (**Figure 3B**) followed a similar trend. At 10% LL, flavonoid levels increased by 27.9% in wheat, 12.1% in barley, 7.1% in rapeseed, and 12.3% in mung bean compared to controls. The 15% LL treatment led to further increases of 49.2% in wheat, 19.8% in barley, 12.0% in rapeseed, and 46.3% in mung bean.

### 3.5 Hydrogen peroxide and lipid peroxidation

**Figure 4:**
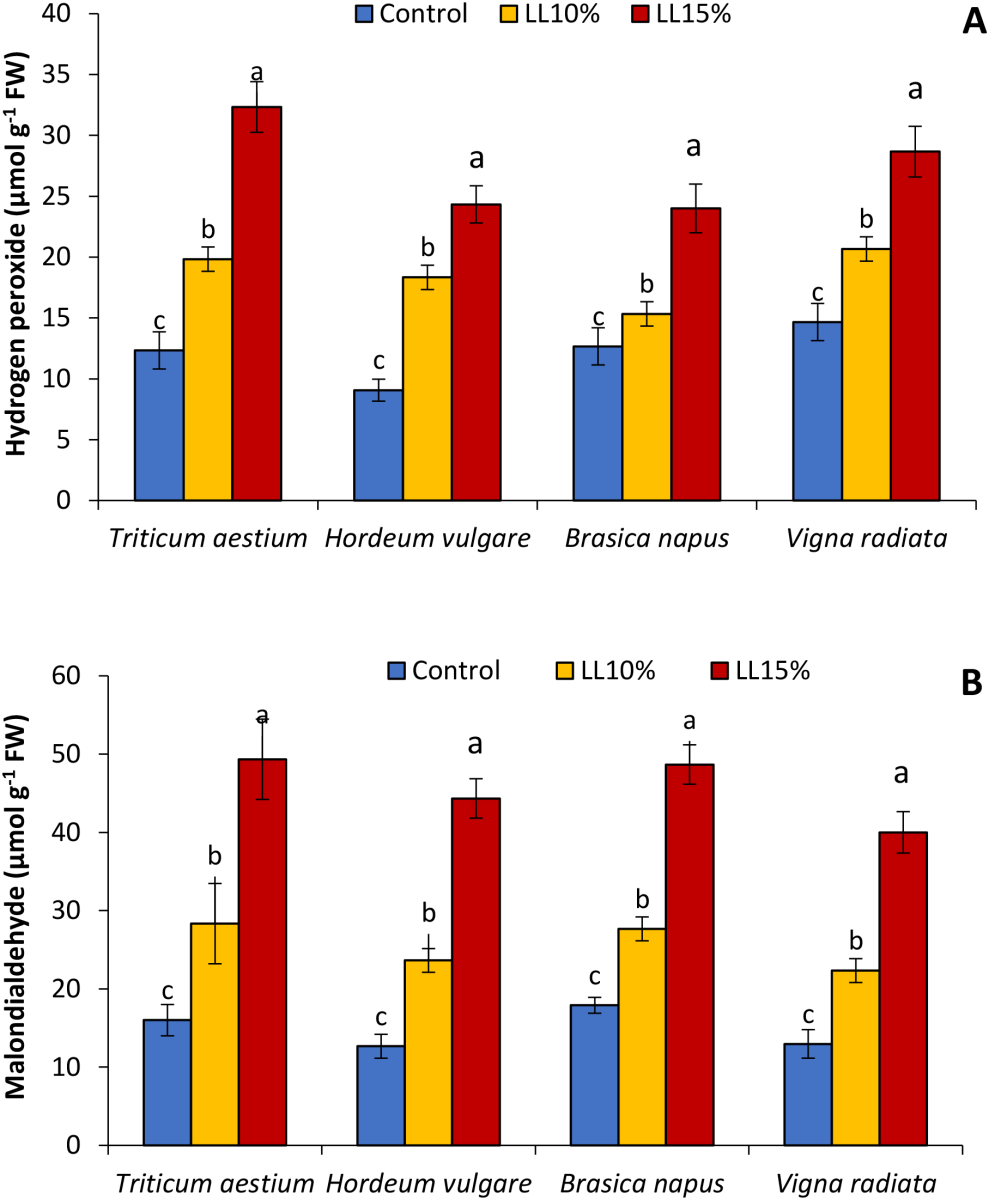
Effect of different concentrations (10% and 15%) of *Aizoon canariense* leaf leachate (LL) on **(A)** hydrogen peroxide and **(B)** malondialdehyde content in wheat (*Triticum aestivum*), barley (*Hordeum vulgare*), rapeseed (*Brassica napus*), and mung bean (*Vigna radiata*). Data represent the mean ± standard error (SE) of three independent replicates. Data represent the means of three replications. Means sharing the same letter are not significantly different at the 5% probability level according to the Least Significant Difference (LSD) test.

The levels of H_2_O_2_ and MDA key indicators of oxidative stress and lipid peroxidation, were significantly affected by *A. canariense* LL treatments in all tested crop species (**Figure 4**). As shown in **Figure 4A**, H_2_O_2_ content increased progressively with LL concentration. Under 10% LL, H_2_O_2_ levels increased by 60.9% in wheat, 102.2% in barley, 21.1% in rapeseed, and 40.9% in mung bean compared to their respective controls. The 15% LL treatment led to even greater increases: 162.2% in wheat, 168.4% in barley, 89.4% in rapeseed, and 95.5% in mung bean, indicating intensified ROS generation under allelopathic stress, particularly in barley and wheat. Similarly, MDA content (**Figure 4B**), which reflects lipid peroxidation and membrane damage, was significantly elevated following LL application. Compared to control values, MDA increased by 77.1% in wheat, 86.5% in barley, 54.5% in rapeseed, and 72.2% in mung bean under the 15% LL treatment. At 10% LL, the relative increases were 77.1% in wheat, 86.5% in barley, 54.5% in rapeseed, and 72.2% in mung bean.

### 3.6 Antioxidant enzyme activities and DPPH scavenging

**Figure 5:**
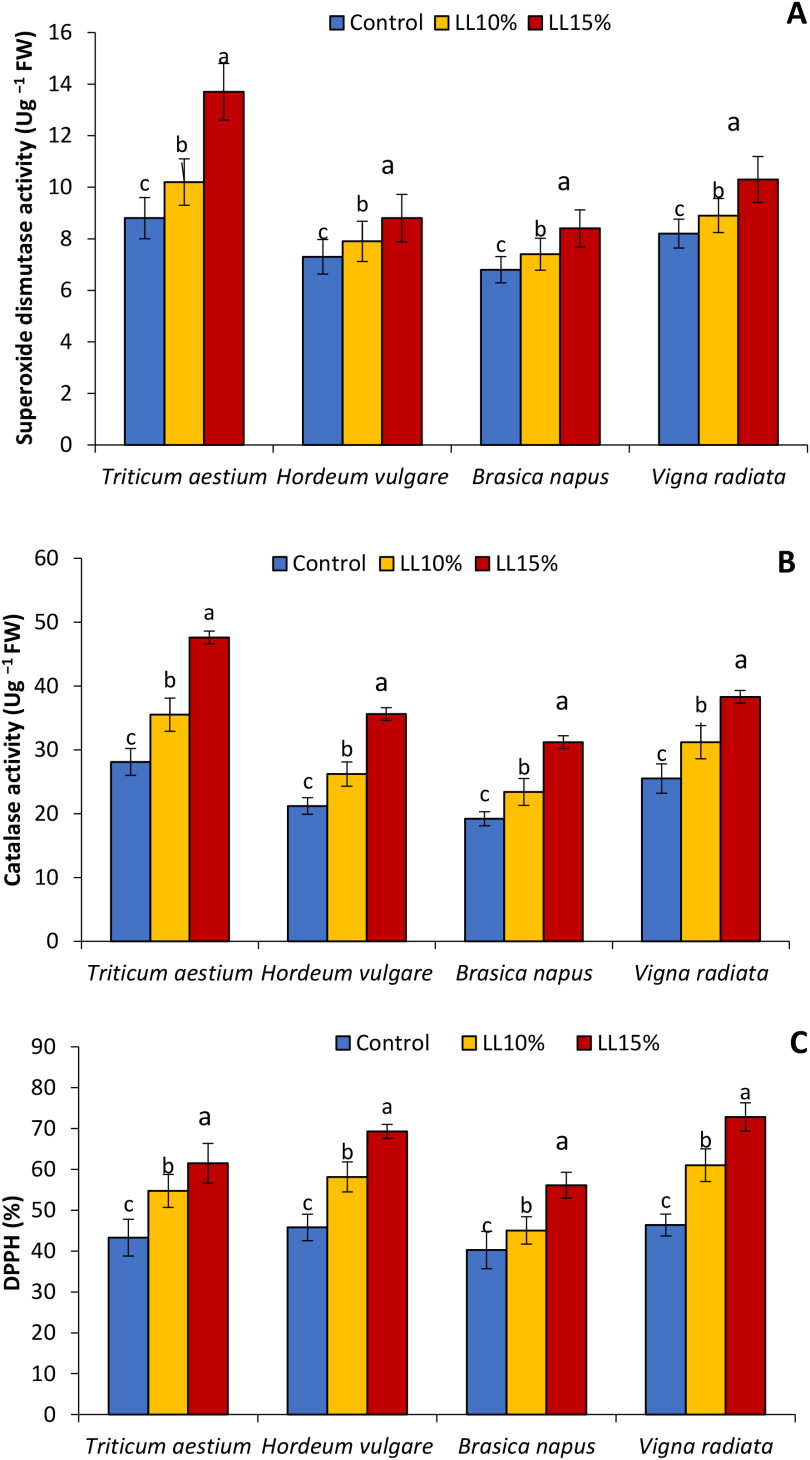
Effect of different concentrations (10% and 15%) of *Aizoon canariense* leaf leachate (LL) on **(A)** superoxide dismutase activity **(B)** catalase activity and **(C)** DPPH in wheat (*Triticum aestivum*), barley (*Hordeum vulgare*), rapeseed (*Brassica napus*), and mung bean (*Vigna radiata*). Data represent the mean ± standard error (SE) of three independent replicates. Data represent the means of three replications. Means sharing the same letter are not significantly different at the 5% probability level according to the Least Significant Difference (LSD) test.

The effect of *A. canariense* LL at 10% and 15% concentrations on antioxidant enzyme activities—SOD and CAT—and DPPH radical scavenging activity in wheat, barley, rapeseed, *Hordeum vulgare*, *Brassica napus*, and *Vigna radiata* is illustrated in **Figure 5**. SOD activity (**Figure 5A**) increased progressively with LL concentration across all species. At 15% LL, SOD activity rose by 55.7% in wheat, 20.5% in barley, 23.5% in rapeseed, and 25.6% in *V. radiata*, indicating a strong activation of antioxidant defenses, especially in wheat.

Similarly, CAT activity (**Figure 5B**) was enhanced in a concentration-dependent manner. The highest relative increases under 15% LL were observed in wheat (69.4%), followed by barley (67.9%), rapeseed (62.5%), and mung bean (50.2%). These elevations suggest that LL-induced oxidative stress stimulates enzymatic antioxidant systems, with the response magnitude varying by species. DPPH scavenging activity (**Figure 5C**), a measure of non-enzymatic antioxidant capacity, also improved significantly. At 15% LL, mung bean and barley showed the highest increases—57.1% and 51.3%, respectively—while wheat and rapeseed recorded increases of 41.9% and 39.5%, respectively.

### 3.7 Correlation analysis

**Figure 6:**
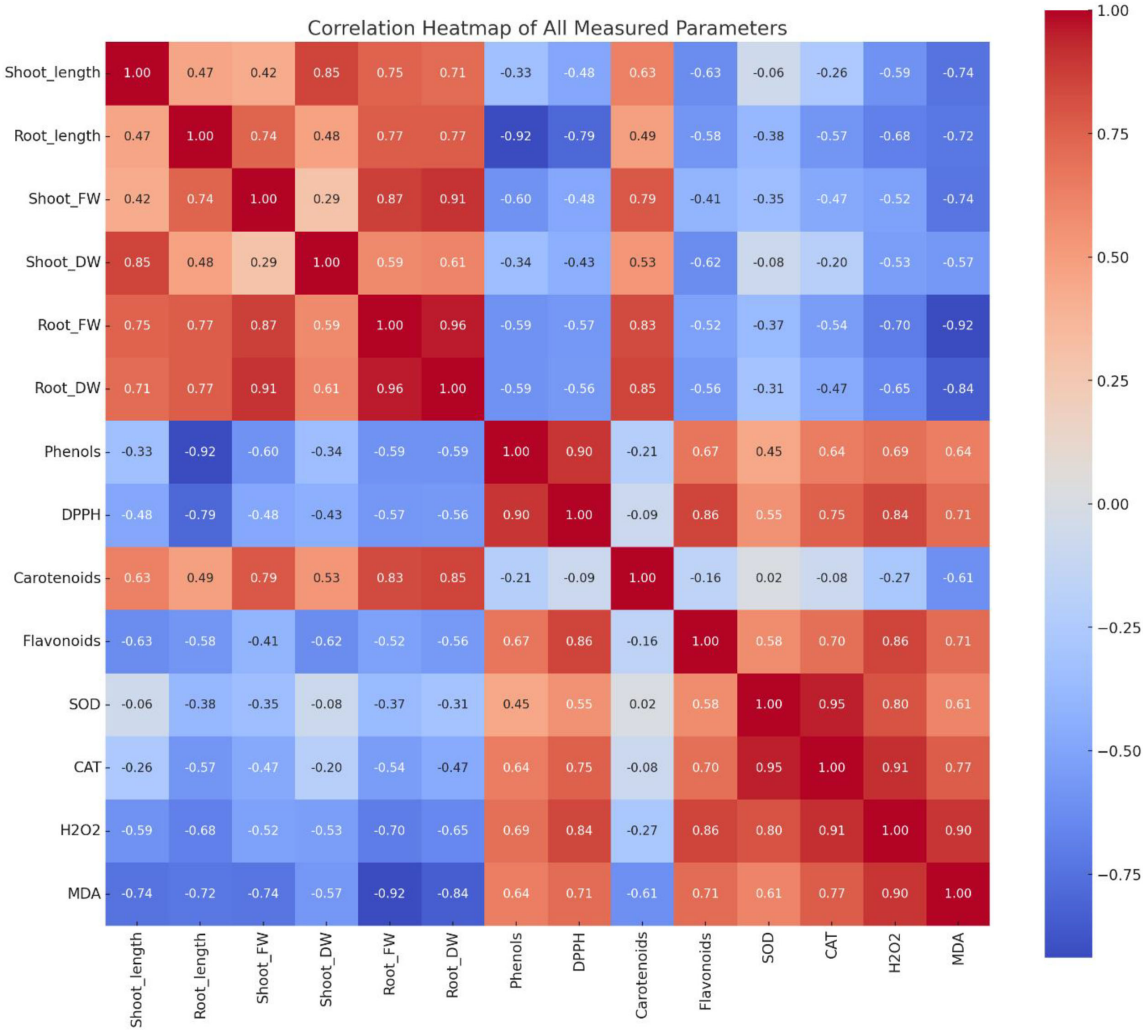
Correlation heatmap showing relationships among growth, antioxidant, and stress-related parameters in crops treated with *Aizoon canariense* leaf leachate.

Correlation analysis revealed distinct relationships between morphological, biochemical, and oxidative stress markers across all plant species and treatments with *A. canariense* leaf leachate (LL). Growth parameters, including shoot length, root length, and biomass components, exhibited strong positive correlations with each other (shoot fresh weight and root dry weight, *r* > 0.90), indicating coordinated growth responses under control conditions (**Figure 6**).

Phenolic content showed a strong positive correlation with DPPH scavenging activity (*r* = 0.90), suggesting that phenolic compounds contributed significantly to the antioxidant activity. Similarly, flavonoid content positively correlated with antioxidant enzymes such as SOD (*r* = 0.58) and CAT (*r* = 0.70), indicating their role in stress mitigation. In contrast, oxidative stress indicators— H_2_O_2_ and MDA—were negatively correlated with growth parameters (MDA vs. shoot length, *r* = –0.74), but positively correlated with SOD, CAT, and phenolic compounds, suggesting that allelopathic stress induced by LL triggered oxidative damage and activated antioxidant defenses. These correlations demonstrate that higher LL concentrations impair plant growth through oxidative stress, which is partially countered by increased antioxidant enzyme activity and secondary metabolite accumulation.

## 4 Discussion

The allelopathic activity of *A. canariense* leaf leachate appears to be driven by its diverse suite of bioactive metabolites identified via GC–MS. Dominant sesquiterpene alcohols such as patchouli alcohol and α-cadinol, along with other sesquiterpenes including e-cadinene and caryophyllene, are well-documented to disrupt membrane integrity, impair ion transport, and promote reactive oxygen species (ROS) generation in target plants (Kumar et al., 2024). Long-chain alkanes like tetratriacontane and dotriacontane can further reduce cuticular permeability and interfere with gas exchange, while chlorophyll degradation products such as phytol may damage chloroplast structure and hinder pigment biosynthesis (Hua et al., 2022). Phenolic-related compounds and cyclic hydrocarbons (e.g., naphthalene derivatives, aromadendrene) are known to inhibit key enzymes in photosynthesis and primary metabolism, potentially diverting resources towards secondary metabolite production and defense — a metabolic trade-off reported in plants under oxidative stress (Hua et al., 2022). The combined phytochemical profile is consistent with the physiological responses observed in treated plants, including enhanced SOD and CAT activities, elevated phenolic and flavonoid contents, and pigment loss. Collectively, these results support a mechanistic sequence in which allelochemicals induce ROS overproduction, activate antioxidant defenses, and alter carbon allocation, culminating in reduced growth and biomass.

The observed decline in chlorophyll and carotenoid content in LL-treated plants is a primary indicator of photosynthetic impairment. Chlorophyll, the central pigment in light-harvesting complexes, is directly linked to photosynthetic rate and biomass accumulation (Simkin et al., 2022). Carotenoids, in turn, serve both as accessory pigments and ROS quenchers that protect the photosystems under stress (Pérez-Gálvez et al., 2020). The concentration-dependent reduction in both pigments suggests that allelochemicals in *A. canariense* LL may disrupt pigment biosynthesis or accelerate pigment degradation via oxidative processes, a phenomenon reported in plants exposed to phenolic- and terpene-rich leachates (Cheng and Cheng, 2015; Pattanayak and Maiti, 2025).

The loss of photosynthetic pigments not only compromises light absorption but also disrupts key metabolic processes such as energy generation, carbon assimilation, and the biosynthesis of defense-related metabolites (Didaran et al., 2024). This pigment degradation is closely associated with the observed reductions in shoot and root growth, as photosynthetic inhibition limits carbohydrate production and thus the energy supply needed for cell expansion and biomass accumulation (Zhang et al., 2020). Importantly, GC-MS analysis of the *A. canariense* leaf leachate revealed the presence of allelochemicals such as patchouli alcohol and phytol, which have been previously shown to impair membrane integrity and modulate redox signaling. Patchouli alcohol, in particular, is known to destabilize lipid bilayers and enhance membrane permeability, resulting in electrolyte leakage and enhanced ROS production (Hua et al., 2022). This oxidative stress, in turn, activates the plant’s antioxidant defense system, as evidenced by the elevated activities of enzymes such as SOD, CAT, and POD in treated seedlings. While these findings support a mechanistic link between allelochemical exposure and redox imbalance, it is important to acknowledge that the current study is limited to seedling-stage responses under controlled conditions.

Interestingly, the application of LL also led to a significant increase in phenolic and flavonoid contents, particularly in rapeseed and mung bean, which were also the most sensitive to pigment loss and growth suppression. Phenolics and flavonoids are well-known ROS scavengers and modulators of signaling pathways under stress conditions (Patil et al., 2024; Zahra et al., 2024). Their accumulation in response to LL treatment suggests a metabolic shift in plants toward the synthesis of protective secondary metabolites, possibly at the expense of growth-related primary metabolism.

This shift reflects an adaptive reprogramming of cellular metabolism, where resources are redirected to bolster antioxidant activity and membrane protection. While beneficial in the short term, this trade-off can inhibit overall growth and development, as evidenced by the strong negative correlation between phenolic/flavonoid levels and growth metrics. This metabolic diversion is a typical response in plants experiencing allelopathic stress, wherein activation of phenylpropanoid and shikimate pathways is favored to counteract oxidative damage.

The elevated levels of SOD and CAT activity further confirm the presence of oxidative stress in LL-treated plants. These enzymes represent the first line of defense against ROS accumulation: SOD catalyzes the dismutation of superoxide radicals to H_2_O_2_, while CAT decomposes H_2_O_2_ into water and oxygen. Their upregulation, particularly at 15% LL, indicates an oxidative burst likely triggered by allelochemicals such as α-cadinol, patchoulol, and phytol—compounds known for their redox-modulating and membrane-disrupting effects (Casanova et al., 2023; Cruz-Ortega et al., 2007).

The increase in enzymatic activity aligns with elevated levels of H_2_O_2_, a signaling molecule that accumulates during abiotic stress and may function to induce further antioxidant defenses (Aqaei et al., 2020; Weisany and Khosropour, 2023). However, excessive H_2_O_2_ can lead to cellular damage, as suggested by the parallel rise in MDA, a product of lipid peroxidation (Li Pomi et al., 2025; Manvelian et al., 2021). MDA accumulation is a robust indicator of membrane damage and correlates strongly with growth inhibition in this study, especially in rapeseed and mung bean. This indicates that, although antioxidant defenses are activated, they may not be sufficient to fully neutralize ROS at higher LL concentrations.

Integrating these findings reveals a clear mechanistic link between pigment loss, ROS generation, secondary metabolite production, and growth inhibition. The application of *A. canariense* LL appears to initiate a cascade of stress responses beginning with disruption of chlorophyll biosynthesis or function. This likely leads to photooxidative stress due to impaired light energy dissipation, triggering overproduction of ROS, especially H_2_O_2_ (Rao et al., 2025). In response, plants attempt to mitigate oxidative damage through the upregulation of antioxidant enzymes (SOD and CAT) and synthesis of phenolic and flavonoid compounds (Rao et al., 2025). However, when the level of oxidative stress exceeds the buffering capacity of these systems—particularly under 15% LL treatment—cellular damage, including lipid peroxidation, ensues. This is reflected in reduced root and shoot elongation and overall biomass decline.

The strong negative correlations between MDA/H_2_O_2_ and growth parameters, and the positive associations between phenolics, flavonoids, and DPPH activity, underscore this interconnected physiological framework. It is evident that the allelochemicals in *A. canariense* LL not only inhibit primary metabolism but also invoke a costly defensive reconfiguration in recipient plants, leading to trade-offs between growth and survival.

While all tested crop species exhibited clear stress responses to *A. canariense* leaf leachate, the extent of inhibition varied considerably, with rapeseed and mung bean displaying greater sensitivity compared to wheat and barley. This interspecific variation in allelopathic response may be attributed to multiple physiological and biochemical factors, including differences in root surface area and exudate absorption rates, variations in membrane permeability, species-specific detoxification mechanisms, and the inherent strength of each species’ antioxidant defense systems (Inderjit and Duke, 2003). From an ecological perspective, these findings are particularly significant as they highlight the potential of *A. canariense* to exert selective allelopathic pressure in mixed plant communities. Such differential susceptibility may influence species composition, competitive hierarchies, and successional dynamics in natural and agro-ecological systems where *A. canariense* is prevalent.

The dose–dependent inhibitory effects of *A. canariense* leaf leachates align with allelopathic patterns observed in other xerophytic species. For instance, *Xanthium strumarium* exhibited concentration-dependent suppression of seedling growth in crops like soybean and wheat at similar extract concentrations (Hassanpouraghdam, 2010), while *Leptochloa chinensis* demonstratedf significant reductions in root elongation and biomass in rice seedlings under increasing leachate doses (Ishfaq et al., 2025). Compared to these species, *A. canariense* showed comparable or even greater phytotoxicity, especially at the 15% concentration, highlighting its potential ecological impact and relevance in arid or semi-arid ecosystems where such xerophytes may influence interspecific competition and community dynamics.

The allelopathic effects observed in this study suggest that *A. canariense* leaf leachates function as potential natural growth modulators capable of suppressing early seedling development in selected crop species. However, while the inhibitory effects—particularly on rapeseed and mung bean—are noteworthy, further research is necessary to substantiate their application in sustainable weed management. Future studies should include soil incubation experiments to assess compound stability and persistence, field trials to evaluate efficacy under natural environmental conditions, and ecotoxicological assays on non-target organisms to ensure ecological safety. Such investigations will help clarify the practical potential of *A. canariense* extracts as part of integrated, eco-friendly weed control strategies and expand our understanding of species-specific interactions within plant communities.

## 5 Conclusions and future directions

*Aizoon canariense* leaf leachates markedly suppressed plant growth by reducing photosynthetic pigments, increasing reactive oxygen species, and impairing antioxidant defenses. At 15% concentration, LL reduced shoot biomass by nearly 50% in rapeseed and mung bean, confirming strong species-specific phytotoxicity. GC–MS analysis implicated sesquiterpenes and diterpenes as key allelochemicals likely driving oxidative stress and membrane damage. These findings highlight the potential of *A. canariense* as a source of natural compounds for sustainable weed management. Future work should isolate and test individual bioactive constituents via bioassay-guided fractionation and evaluate their selectivity, persistence, and environmental safety through soil-based and field trials under agronomic conditions.

## Data Availability

The raw data supporting the conclusions of this article will be made available by the authors, without undue reservation.
